# Comparison of effective regurgitant orifice area by the PISA method and tricuspid coaptation gap measurement to identify very severe tricuspid regurgitation and stratify mortality risk

**DOI:** 10.3389/fcvm.2023.1090572

**Published:** 2023-04-27

**Authors:** Yohann Bohbot, Léa Tordjman, Julien Dreyfus, Thierry Le Tourneau, Yoan Lavie-Badie, Christine Selton-Suty, Benjamin Elegamandji, Guillaume L’official, Antoine Fraix, Samy Aghezzaf, Pierre Yves Turgeon, David Messika Zeitoun, Maurice Enriquez-Sarano, Augustin Coisne, Erwan Donal, Christophe Tribouilloy

**Affiliations:** ^1^ Department of Cardiology, Amiens University Hospital, Amiens, France; ^2^ UR UPJV 7517, Jules Verne University of Picardie, Amiens, France; ^3^ Cardiology Department, Centre Cardiologique du Nord, Saint-Denis, France; ^4^ l'institut du thorax, INSERM, CNRS, UNIV Nantes, Nantes, France; ^5^ Department of Cardiology, Rangueil University Hospital, Toulouse, France; ^6^ Cardiology Department CIC-EC, CHU Nancy-Brabois, Nancy, France; ^7^ CHU Rennes, Inserm, LTSI—UMR 1099, University of Rennes, Rennes, France; ^8^ Inserm, CHU Lille, Institut Pasteur de Lille, U1011—EGID, University Lille, Lille, France; ^9^ Department of Cardiology, University of Ottawa Heart Institute, Ottawa, ON, Canada; ^10^ Valve Science Center, Minneapolis Heart Institute Foundation, Minneapolis, MN, United States; ^11^ Cardiovascular Research Foundation, New York, NY, United States

**Keywords:** very severe tricuspid regurgitation, survival, coaptation gap, effective regurgitant orifice area, mortality

## Abstract

**Introduction:**

Various definitions of very severe (VS) tricuspid regurgitation (TR) have been proposed based on the effective regurgitant orifice area (EROA) or tricuspid coaptation gap (TCG). Because of the inherent limitations associated with the EROA, we hypothesized that the TCG would be more suitable for defining VSTR and predicting outcomes.

**Materials and methods:**

In this French multicentre retrospective study, we included 606 patients with ≥moderate-to-severe isolated functional TR (without structural valve disease or an overt cardiac cause) according to the recommendations of the European Association of Cardiovascular Imaging. Patients were further stratified into VSTR according to the EROA (≥60 mm^2^) and then according to the TCG (≥10 mm). The primary endpoint was all-cause mortality and the secondary endpoint was cardiovascular mortality.

**Results:**

The relationship between the EROA and TCG was poor (*R*^2 ^=^ ^0.22), especially when the size of the defect was large. Four-year survival was comparable between patients with an EROA <60 mm^2^ vs. ≥60 mm^2^ (68 ± 3% vs. 64 ± 5%, *p* = 0.89). A TCG ≥10 mm was associated with lower four-year survival than a TCG <10 mm (53 ± 7% vs. 69 ± 3%, *p* < 0.001). After adjustment for covariates, including comorbidity, symptoms, dose of diuretics, and right ventricular dilatation and dysfunction, a TCG ≥10 mm remained independently associated with higher all-cause mortality (adjusted HR[95% CI] = 1.47[1.13–2.21], *p* = 0.019) and cardiovascular mortality (adjusted HR[95% CI] = 2.12[1.33–3.25], *p* = 0.001), whereas an EROA ≥60 mm^2^ was not associated with all-cause or cardiovascular mortality (adjusted HR[95% CI]: 1.16[0.81–1.64], *p* = 0.416, and adjusted HR[95% CI]: 1.07[0.68–1.68], *p* = 0.784, respectively)

**Conclusion:**

The correlation between the TCG and EROA is weak and decreases with increasing defect size. A TCG ≥10 mm is associated with increased all-cause and cardiovascular mortality and should be used to define VSTR in isolated significant functional TR.

## Introduction

Tricuspid regurgitation (TR) is a frequent valvular condition that affects approximately 0.8% of the general population (1). Its prevalence increases significantly in elderly patients, especially women, and in cases of atrial fibrillation (AF) (2). Although mild TR is generally considered to be benign, severe TR is systematically associated with a poor prognosis (3–6). Recent data also suggest that even moderate TR is associated with an adverse outcome (7, 8). Therefore, accurate quantification of TR severity by Doppler echocardiography is essential. Over the past few years, with the development of transcatheter therapies, the concept of very severe tricuspid regurgitation (VSTR) has emerged (9, 10). Massive and torrential TR were thus added to the TR grading scheme using empirical thresholds for effective regurgitant orifice area (EROA) measurements (11). Subsequently, a study reported that patients with ≥massive TR, using this definition, experienced greater mortality than those with “only severe” TR, with no difference between massive and torrential forms (12). Thus, the distinction between massive and torrential TR does not appear to be relevant for risk stratification in clinical practice, but a grade in addition to severe TR (very severe TR, VSTR) had to unquestionably be added to the classification.

Various definitions of VSTR have been proposed since, using EROA or tricuspid coaptation gap (TCG) (13–15). Because of the inherent limitations associated with the EROA (16), the definition of VSTR is a matter of debate. We hypothesized that the TCG would be more suitable for defining VSTR and predicting outcomes. Therefore, we compared the EROA, measured by the PISA method, and the TCG measurement to identify VSTR and stratify mortality risk in a multicentre French cohort of patients with isolated functional TR.

## Materials and methods

### Study protocol

This retrospective study was conducted in seven French tertiary centers (Amiens, Lille, Nancy, Nantes, Rennes, Saint Denis, and Toulouse) and included consecutive patients with a diagnosis of significant (≥moderate-to-severe) isolated TR between 2013 and 2020. Isolated functional TR was defined by structurally normal tricuspid valves with no overt organic cause of TR, no >mild left-sided valvular heart disease, no pulmonary hypertension, preserved (>50%) left ventricular ejection fraction (LVEF), and no previous cardiac surgery (17). Baseline clinical and demographic characteristics were collected. The Charlson comorbidity index, comprising the sum of individual comorbidities, was calculated for each patient (18). This study was conducted in accordance with local institutional policies, French legislation, and the revised Declaration of Helsinki and was approved by each local institutional review board. The data underlying this article will be shared upon reasonable request to the corresponding author.

### Echocardiography

A complete evaluation by Doppler echocardiography was performed on all patients by experienced echocardiographers using commercially available ultrasound systems. TR was graded as moderate-to-severe and severe by the principal investigator of each center based on the European association of cardiovascular imaging (EACVI) recommendations using the integrative multiparametric approach, including morphological (tricuspid valve morphology, aspect of TR colour flow and continuous Doppler jet), semi quantitative (vena contracta, PISA radius, hepatic vein flow and tricuspid inflow), and quantitative (EROA and regurgitant volume) parameters (19). Patients were reclassified as VSTR based on an EROA ≥60 mm^2^ (11) and then a TCG ≥10 mm. The PISA radius was measured in mid-systole using a color baseline shift between 20 and 40 cm/s in the regurgitation direction with a zoom to the convergence zone (19–21) but the ratio of the aliasing velocity to the orifice jet velocity was maintained, as recommended, <10% whenever feasible. EROA correction for leaflet angles was performed by clinicians when the angle appeared much larger than 180°. The tricuspid coaptation gap was evaluated by 2D echocardiography and measured in end-systole in each available view to identify the largest defect of coaptation. The size of the gap between leaflets was measured at the tip of the leaflets. RV assessment was performed on multiple views. RV function was assessed using M-mode analysis of the tricuspid annulus systolic excursion (TAPSE) and *S*′ wave velocity at the lateral tricuspid annulus obtained by pulsed wave tissue Doppler imaging. RV dilation was defined by a RV basal diameter >42 mm in an apical four-chamber view (22). Moderate/severe RV dysfunction was defined by either a TAPSE <14 mm and/or an *S*′ < 10 cm/s (22). Tricuspid annulus diameter was measured in diastole in four-chamber view. Low flow was defined by a stroke volume <46 ml for women and <51 ml for men (23).

### Follow-up and endpoints

During follow-up, events were collected based on hospital chart reviews, direct patient interviews and/or repeated follow-up letters, questionnaires, and telephone calls to physicians, patients, and (if necessary) family members. The primary endpoint was all-cause mortality and the secondary endpoint cardiovascular mortality, including sudden death, Heart failure related death and deaths attributable to other cardiovascular causes (such as myocardial infarction, stroke…). Decisions on medical or surgical management were made by each heart team based on European Society of Cardiology guidelines and patient comorbidities (10).

### Statistical analysis

Baseline continuous variables are expressed as means ± standard deviations and categorical variables as numbers and percentages. The differences between the three groups (moderate-to-severe TR, severe TR, and VSTR) based on baseline continuous variables were explored using the Student *t* test. The *χ*^2^ or Fisher exact test were used to compare categorical variables between groups. The significance between the referent group (moderate-to-severe TR) and the other groups was examined when a significant difference between categories was observed. Individual differences were compared using Mann–Whitney *U*-tests (with a Bonferroni correction for multiple comparisons) and Tukey tests for normally distributed data. The presence of VSTR was assessed in each centre, prior to that study, by the physician's overall judgement of the regurgitation severity and consequences. After integration of all these parameters, the echocardiographer graded TR as severe or VSTR. Using ROC curve analysis, we compared the ability of the TCG and the EROA to diagnose VSTR as defined above. The correlation between EROA and TCG was tested by linear regression. Interobserver reproducibility for EROA and TCG measurements was evaluated using Pearson correlation coefficient and intraclass correlation coefficient (ICC) analysis using a randomly chosen subset of 20 patients with severe TR. Event rates ± standard errors were estimated using the Kaplan-Meier method and compared by two-sided log-rank tests. Multivariable analyses for all-cause and cardiovascular mortality in the overall study population and in patients with a tricuspid coaptation gap ≥10 mm, were performed using Cox proportional hazard models. Clinical and echocardiographic covariates considered to have a potential prognostic impact were tested in univariate analysis. All significant variables in univariate analysis with *p* < 0.1 were included in the multivariate cox analyses. The limit of statistical significance was *p* < 0.05 and all tests were two-tailed. Data were analyzed using SPSS 25.0 (SPSS Inc. Chicago, IL).

## Results

### Comparison of the EROA and TCG to define VSTR

**Figure 1:**
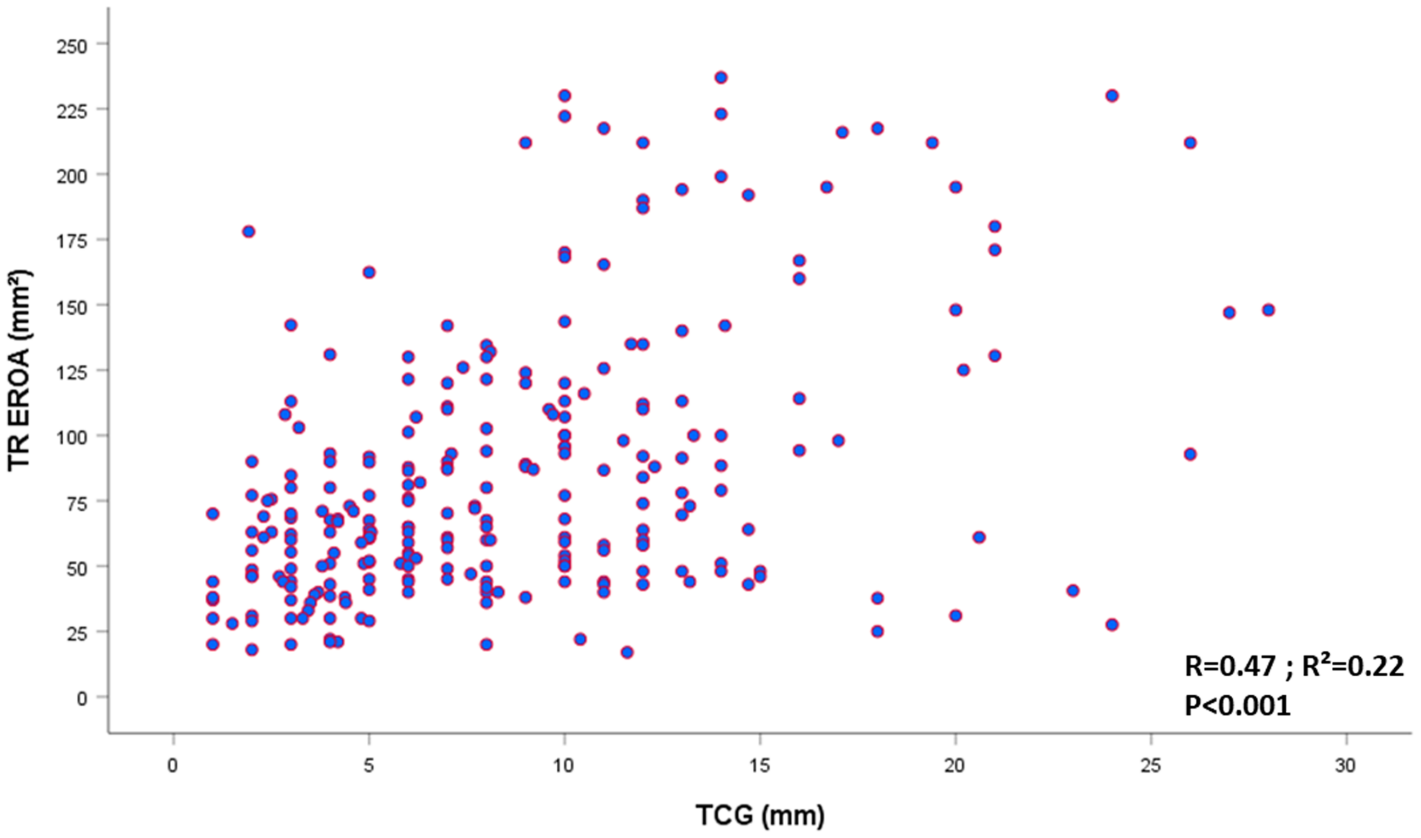
Scatter plot representing the relatioship between TREROA and TCG in patients with visible TCG in 2D (270/606; 45%). EROA, effective regurgitant orifice area; TCG, tricuspid coaptation gap; TR, tricuspid regurgitation.

The study population consisted of 606 patients [234 males (38.6%); mean age: 75 years]. TR was graded, using a multiparametric approach, as moderate-to-severe for 212 patients (35%) and severe for 394 patients (65%). The area under the curve (AUC) to predict VSTR graded using a multiparametric approach was only 0.74 for EROA and 0.95 for TCG (Supplementary Figure S1). A TCG was visible in 270 patients (45%). A TCG of 10 mm (*n* = 104, 17%) was the best cutoff with a sensitivity of 83% and a specificity of 100% to predict VSTR whereas an EROA of 0.6 cm^2^ (*n* = 215, 35%) had a sensitivity of 75% and a specificity of 52% to predict VSTR. Interobserver reproducibility was quite good for the EROA measurement (ICC: 0.89[95% CI: 0.73–0.95), *r* = 0.87), but better for TCG measurement (ICC: 0.95 (95% CI: 0.85–0.98], *r* = 0.92). The correlation between EROA and TCG was poor (*R*^2 ^=^ ^0.22), especially when the size of the defect increased (Figure 1). Among patients with a TCG ≥10 mm (*n* = 104, 17%), 73% (*n* = 76) had an EROA ≥60 mm^2^ and 27% (*n* = 28) an EROA between 40 and 60 mm^2^. Correlations between TCG and basal RV diameter and between TCG and tricuspid annular diameter were weak (*R*^2 ^=^ ^0.17 and 0.19 respectively). In the subset of patients with a TCG and available data on vena contracta (*n* = 197), the correlation between TCG and vena contracta was only moderate (*R* = 0.62; *R*^2 ^=^ ^0.39).

### Baseline clinical characteristics according to TR grade

Baseline characteristics of the patients according to TR grade (moderate-to-severe or severe TR defined using a multiparametric approach, then reclassified as VSTR for a TCG ≥10 mm) are presented in Table 1. Patients with severe and VSTR had more RHF signs, a higher incidence of ascites, used higher doses of loop diuretics, and were more frequently in AF than patients with moderate-to-severe TR (all *p* < 0.05) (Table 1). Patients with VSTR were more likely to have liver cirrhosis (*p* < 0.001) than patients with moderate-to-severe TR (Table 1).

**Table 1:** Baseline characteristics according to TR severity with very severe TR defined using coaptation gap ≥10 mm.

Characteristics	Moderate to severe TR (*N* = 212)	Severe TR (*N* = 290)	Very severe TR (*N* = 104)	*p*-value
**Clinical**				
Age, years	75 ± 13	74 ± 13	75 ± 11	0.582
Female gender (%, *n*)	64.2 (134)	60.3 (175)	58.7 (61)	0.564
Body surface area, m^2^	1.77 ± 0.20	1.81 ± 0.21	1.83 ± 0.21	0.060
Hypertension (%, *n*)	68.4 (145)	59.7 (173)	65.4 (68)	0.122
Diabetes mellitus (%, *n*)	21.7 (46)	25.2 (73)	21.2 (22)	0.565
Liver cirrhosis (%, *n*)	2.4 (5)	4.8 (14)	12.5 (13)^†^	**0**.**001**
Charlson index	3.1 ± 2.3	2.9 ± 2.1	3.2 ± 2.1	0.473
NYHA functional class III–IV (%, *n*)	38.7 (82)	39.7 (115)	40.4 (42)	0.953
Right-sided heart failure signs (%, *n*)	46.2 (98)	59.3 (172)^†^	86.5 (90)^†^	**<0**.**001**
Ascites (%, *n*)	4.2 (9)	8.6 (25)*	14.4 (15)*	**0**.**007**
Loop diuretics (%, *n*)	71.7 (152)	82.1 (238)^†^	87.5 (91)^†^	**0**.**001**
Daily dose of loop diuretics, mg	165 ± 231	164 ± 221	190 ± 231	0.623
Atrial fibrillation (%, *n*)	75.9 (161)	81.4 (236)*	91.3 (95)*	**0**.**004**
**Biological**				
Hemoglobin, g/dl	12.1 ± 2.1	12.2 ± 2.0	11.8 ± 2.0	0.311
Serum creatinine (µmol/L)	111 ± 73	118 ± 95	116 ± 68	0.690
**Echocardiographic**				
LV ejection fraction, %	60 ± 7	59 ± 7	59 ± 7	0.169
LV stroke volume (ml)	66 ± 21	62 ± 21	63 ± 23	0.153
Left atrial area (cm^2^)	27 ± 10	29 ± 10*	31 ± 10*	**0**.**004**
Right atrial area (cm^2^)	28 ± 10	34 ± 11^†^	42 ± 13^†^	**<0**.**001**
TR EROA (mm^2^)	29 ± 7	67 ± 28^†^	115 ± 70^†^	**<0**.**001**
TR *V*_max_ (cm/s)	2.7 ± 0.32	2.5 ± 0.41*	2.25 ± 0.65^†^	**<0**.**001**
TR VTI (cm)	107 ± 38	85 ± 35^†^	72 ± 34^†^	**<0**.**001**
Regurgitant volume (ml)	31 ± 11	55 ± 20^†^	70 ± 35^†^	**<0**.**001**
Laminar TR (%, *n*)	0 (0)	44.1 (90/204)^†^	100 (85/85)^†^	**<0**.**001**
Moderate/severe RV dilatation (%, *n*)	59.9 (127)	79.3 (230)^†^	84.6 (88)^†^	**<0**.**001**
TAPSE, mm	19 ± 4	18 ± 5	19 ± 6	0.508
Peak systolic annular velocity *S*′, cm/s	10.9 ± 2.7	10.9 ± 3.2	10.8 ± 3.3	0.967
RV FAC (%)	41 ± 11	41 ± 11	40 ± 9	0.572
RV free wall strain (%)**	−21 ± 7	−20 ± 7	−22 ± 5	0.173
Tricuspid annulus diameter (mm)	42 ± 6	46 ± 6^†^	51 ± 7^†^	**<0**.**001**
TR peak jet velocity (m/s)	2.9 ± 0.5	2.7 ± 0.6^†^	2.2 ± 0.6^†^	**<0**.**001**
Dilated inferior vena cava (%, *n*)	59.0 (125)	79.0 (229)^†^	95.2 (99)^†^	**<0**.**001**
Dilated subhepatic veins (%, *n*)	25.5 (54)	54.1 (157)^†^	100 (104)^†^	**<0**.**001**
Subhepatic vein flow reversal (%, *n*)	23.9 (43/180)	55.8 (134/240)^†^	100 (80/80)^†^	**<0**.**001**

EROA, effective regurgitant orifice area; FAC, fractional area change; LV, left ventricular, NYHA,  New York Heart Association; RHF, right heart failure; RV, right ventricular; TAPSE, tricuspid annular plane systolic excursion; TR, tricuspid regurgitation; *V*max, peak orifice velocity; VTI, velocity time integral.

Continuous variables are expressed as mean ± 1 standard deviation and categorical variables are expressed as percentages and numbers.

Bold values refers to *p* ≤ 0.05.

^†^ *p* < 0.001 vs. moderate-to-severe TR.

* *p* < 0.05 vs. moderate-to-severe TR.

** Available for 317/606 patients.

In terms of echocardiographic parameters, the area of the left and right atria, TR EROA, and tricuspid annulus diameter increased with TR severity, whereas the TR peak jet velocity decreased. Patients with severe and VSTR were more likely to have inferior vena cava and subhepatic vein dilatation and subhepatic vein flow reversal than patients with moderate-to-severe TR (Table 1).

### Comparison of the EROA and TCG to predict outcome

**Figure 2:**
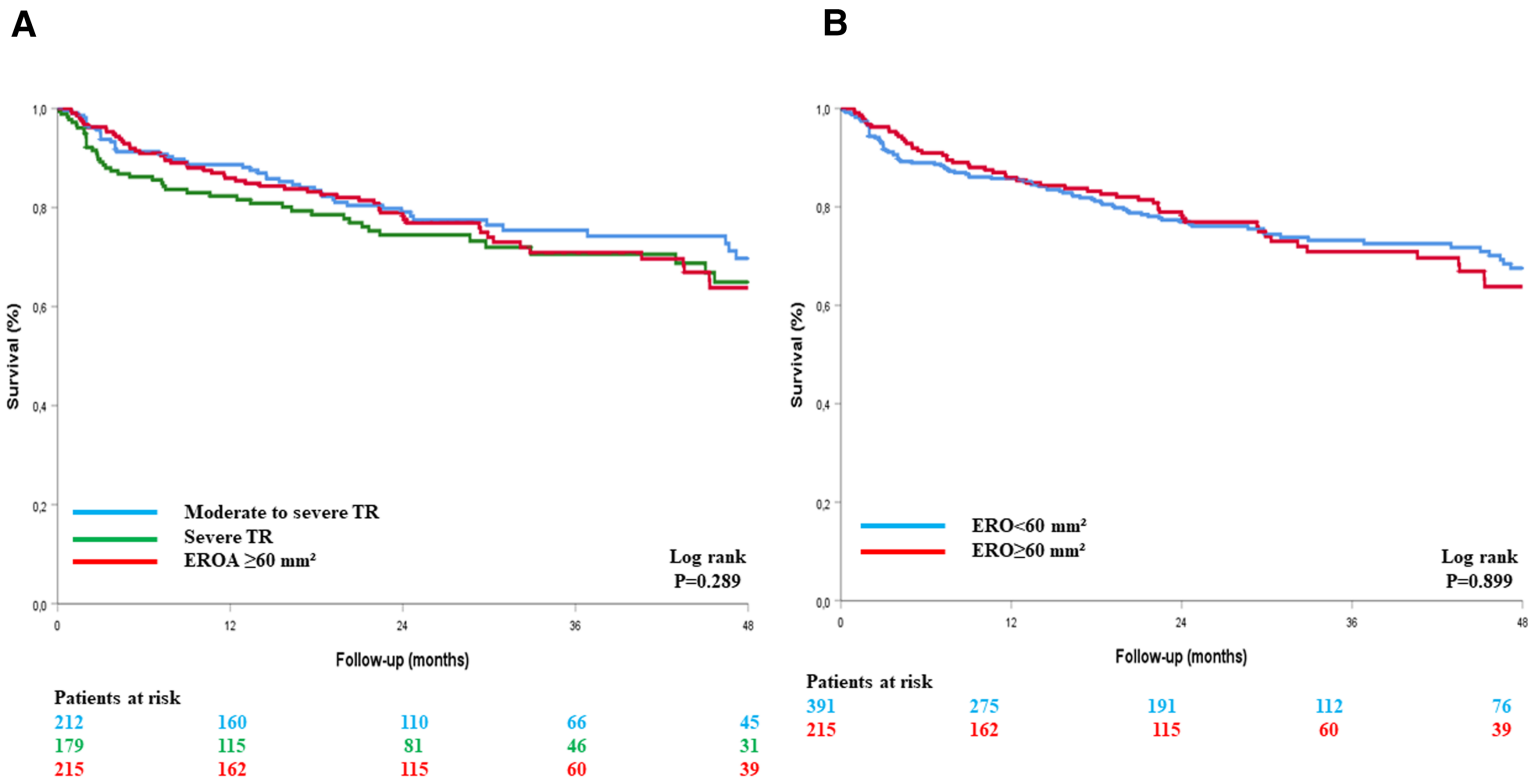
Kaplan–Meier survival curves of patients with TR, with VSTR defined by an EROA ≥60 mm^2^, in three groups (**A**) and in two groups (**B**). EROA, effective regurgitant orifice area; TCG, tricuspid coaptation gap; TR, tricuspid regurgitation.

**Figure 3:**
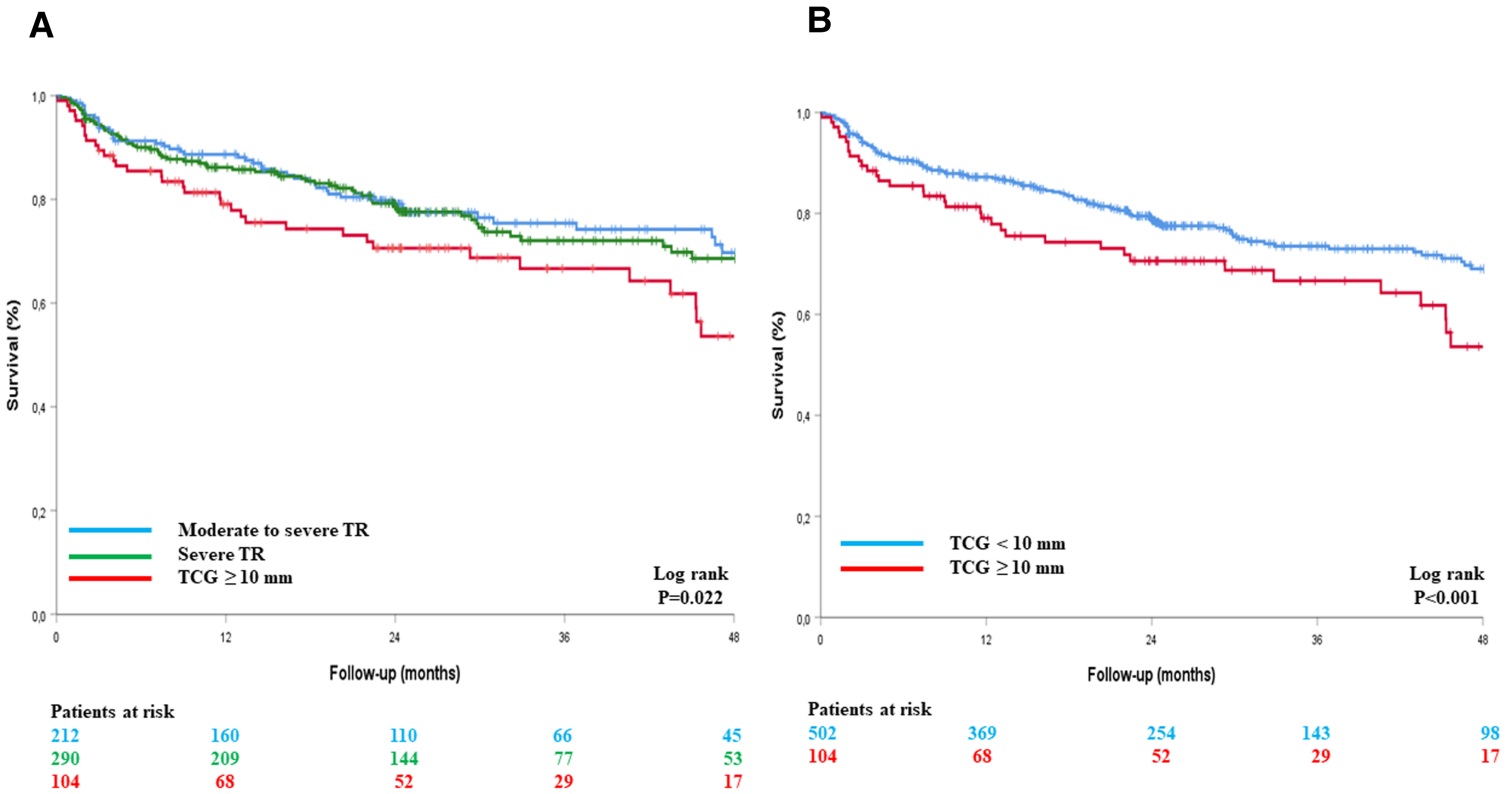
Kaplan–Meier survival curves of patients with TR, with VSTR defined by TCG ≥10 mm, in three groups (**A**) and in two groups (**B**). TCG, tricuspid coaptation gap; TR, tricuspid regurgitation.

In total, 172 (28.4%) deaths occurred during follow-up [median 24 (IQR: 11–41) months, mean 28.5 ± 23 months], of which 100 were attributed to cardiovascular causes (58%). When using an EROA ≥60 mm^2^ to define VSTR, estimated four-year survival rates were 70 ± 4% for moderate-to-severe TR, 65 ± 5% for severe TR, and 64 ± 5% for VSTR (Log rank *p* = 0.289), with no significant difference between patients with an EROA <60 mm^2^ and those with an EROA ≥60 mm^2^ (68 ± 3% vs. 64 ± 5%, respectively, Log rank *p* = 0.899) (Figure 2). When a TCG ≥10 mm was used to define VSTR, estimated four-year survival rates were 70 ± 4% for moderate-to-severe TR, 68 ± 4% for severe TR, and 53 ± 7% for VSTR (Log rank *p* = 0.022), with a significant difference between patients with a TCG <10 mm and those with a TCG ≥10 mm (69 ± 3% vs. 53 ± 7% respectively, Log rank *p* < 0.001) (Figure 3).

**Figure 4:**
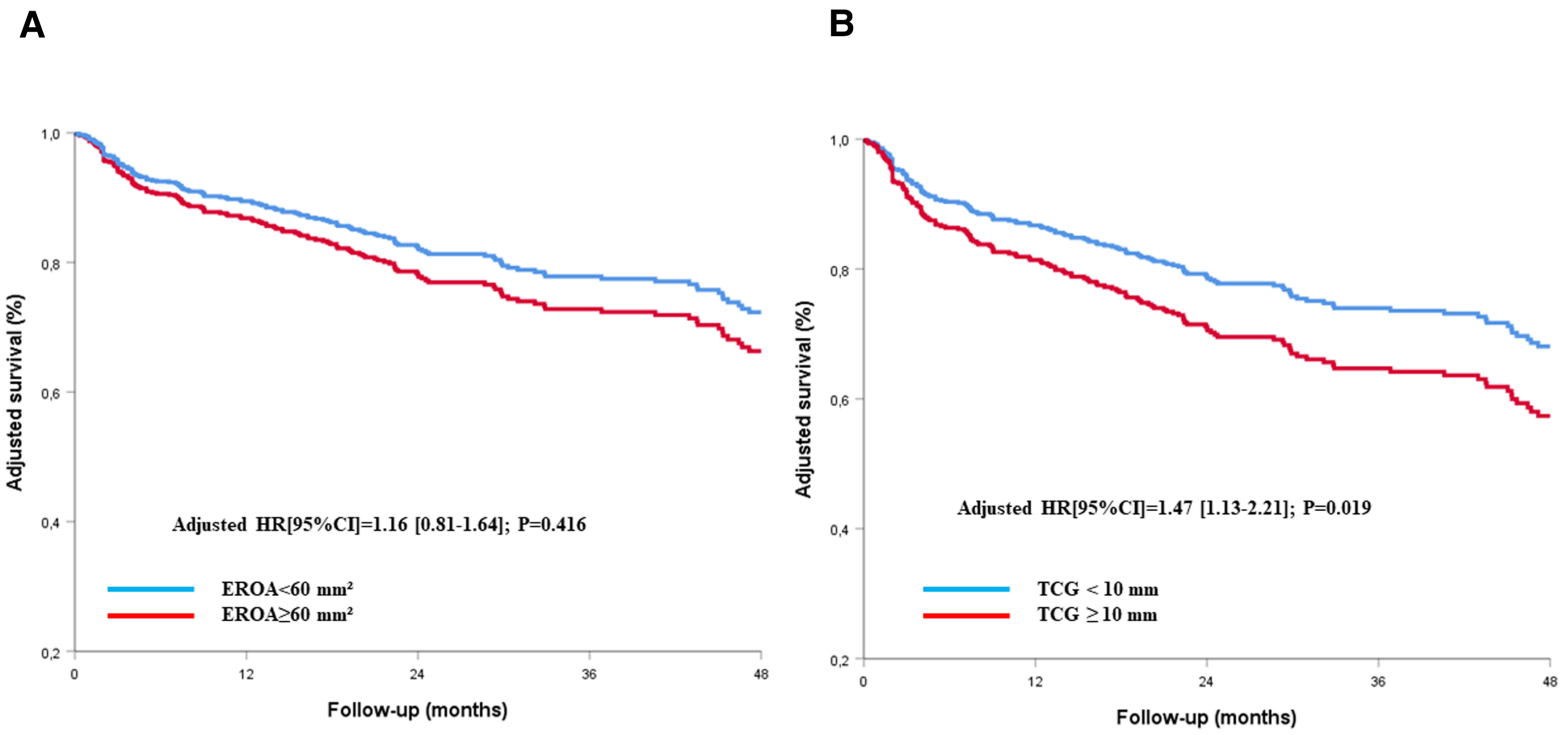
Cox-adjusted survival curves of patients with TR according to the definition of very severe TR using EROA (**A**) or TCG (**B**). EROA, effective regurgitant orifice area; TCG, tricuspid coaptation gap; TR, tricuspid regurgitation.

**Figure 5:**
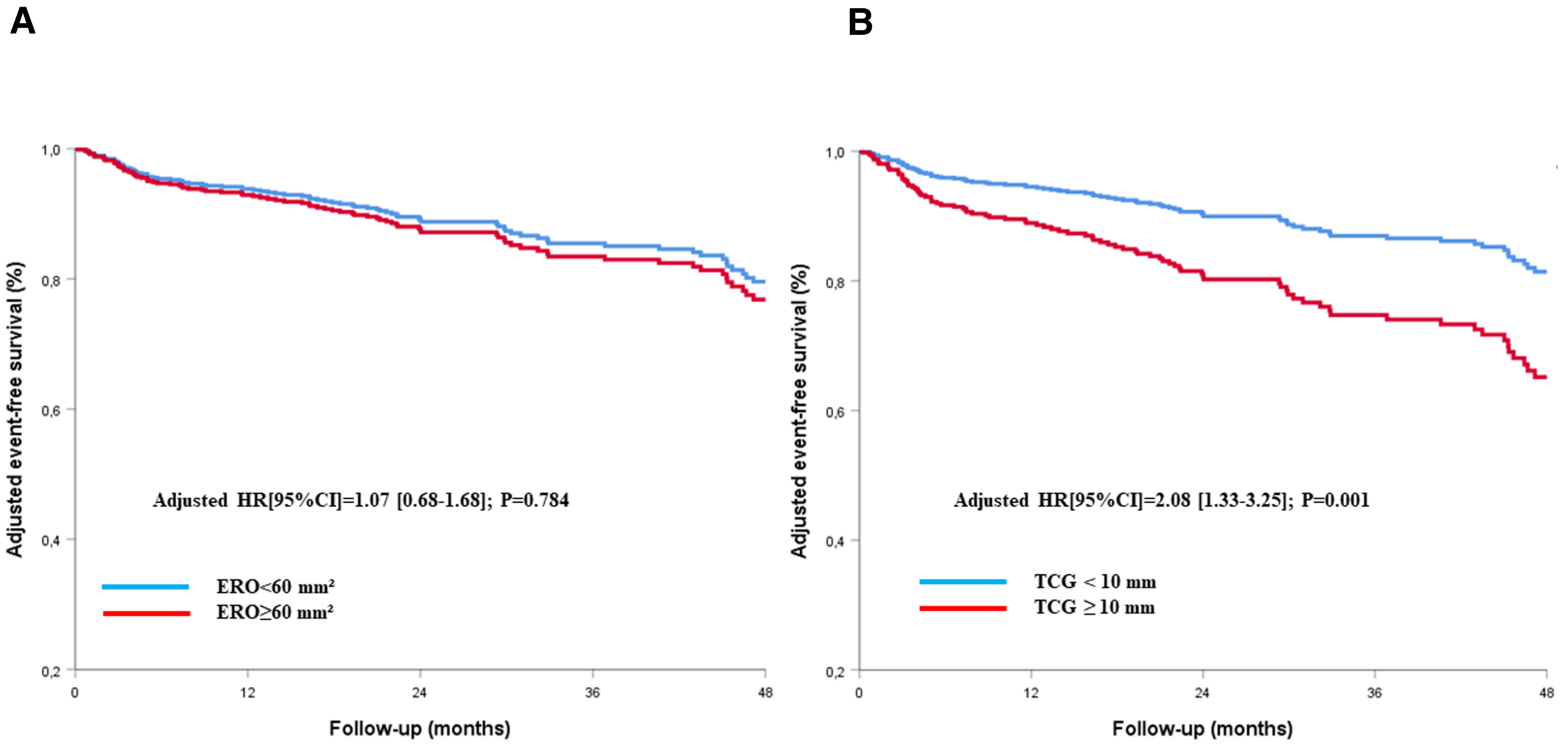
Cox-adjusted curves of freedom from cardiac death for patients with TR according to the definition of very severe TR using EROA (**A**) or TCG (**B**). EROA, effective regurgitant orifice area; TCG, tricuspid coaptation gap; TR, tricuspid regurgitation.

By multivariate analysis, after adjustment for all variables associated with all-cause mortality on univariate analysis (*p* < 0.1, Table 2), the EROA (as a continuous variable) was not associated with all-cause mortality (adjusted HR [95% CI]: 1.00 [0.99–1.01], *p* = 0.186 per mm^2^ increase), whereas the TCG was (adjusted HR [95% CI]: 1.04 [1.01–1.12], *p* = 0.035 per mm increase). After adjustment to the same variables, a TCG ≥10 mm remained independently associated with all-cause mortality (adjusted HR [95% CI]: 1.47 [1.13–2.21], *p* = 0.019), whereas an EROA ≥60 mm^2^ did not (adjusted HR [95% CI]: 1.16 [0.81–1.64], *p* = 0.416) (Table 2, Figure 4). The results were comparable for cardiovascular mortality (adjusted HR [95% CI]: 1.07 [0.68–1.68], *p* = 0.784 for EROA ≥60 mm^2^ and adjusted HR [95% CI]: 2.08 [1.33–3.25], *p* = 0.001 for TCG ≥10 mm) after adjustment for the all variables associated with cardiovascular mortality on univariate analysis (Table 2, Figure 5).

**Table 2:** Clinical and echocardiographic factors associated with all cause and cardiovascular mortality in the study population.

Variable	All-cause mortality				Cardiovascular mortality			
	Univariable analysis HR (CI[95%])	*p*-value	Multivariable analysis HR (CI[95%])	*p*-value	Univariable analysis HR (CI[95%])	*p*-value	Multivariable analysis HR (CI[95%])	*p*-value
Age >75 years	1.33 (0.98–1.82)	0.066	1.45 (1.04–2.00)	**0** **.** **026**	1.18 (0.79–1.76)	0.416	–	–
Male sex	1.41 (1.0–1.90)	**0** **.** **026**	1.41 (1.02–1.95)	**0** **.** **034**	1.68 (1.13–2.49)	**0** **.** **010**	1.51 (0.99–2.30)	0.051
NYHA (3–4 vs. 1–2)	1.82 (1.34–2.45)	**<0** **.** **001**	1.51 (1.09–2.10)	**0** **.** **013**	2.11 (1.42–3.12)	**<0** **.** **001**	1.82 (1.20–2.77)	**0** **.** **005**
Atrial fibrillation	1.25 (1.02–1.84)	**0** **.** **033**	1.08 (0.71–1.65)	0.709	1.18 (0.71–1.95)	0.517	–	–
Right heart failure	1.85 (1.34–2.57)	**<0** **.** **001**	1.19 (0.83–1.71)	0.332	1.21 (0.46–3.18)	0.706	–	–
Ascites	1.92 (1.22–3.00)	**0** **.** **004**	1.61 (0.99–2.60)	0.051	3.03 (1.87–4.90)	**<0** **.** **001**	1.80 (1.04–3.13)	**0** **.** **037**
Charlson comorbidity index >2 (without age)	2.27 (1.64–3.13)	**<0** **.** **001**	1.97 (1.42–2.73)	**<0** **.** **001**	2.11 (1.39–3.22)	**<0** **.** **001**	1.72 (1.13–2.64)	**0** **.** **012**
Diuretics dose ≥125 mg/day	1.95 (1.43–2.67)	**<0** **.** **001**	1.47 (1.05–2.06)	**0** **.** **024**	2.57 (1.74–3.82)	**<0** **.** **001**	1.70 (1.11–2.63)	**0** **.** **016**
LVEF 50%–60%	1.29 (0.95–1.75)	0.097	1.16 (0.83–1.62)	0.382	1.53 (1.03–2.27)	**0** **.** **035**	1.28 (0.84–1.96)	0.249
Low flow	1.44 (1.06–1.94)	**0** **.** **018**	1.44 (1.06–1.96)	**0** **.** **020**	1.63 (1.10–2.42)	**0** **.** **015**	1.50 (1.00–2.25)	**0** **.** **049**
LA area >30 cm^2^	1.24 (0.89–1.76)	0.200	–	–	1.17 (0.58–2.37)	0.664	–	–
RA area >30 cm^2^	1.01 (0.75–1.36)	0.842	–	–	1.72 (1.15–2.57)	**0** **.** **008**	1.16 (0.70–1.93)	0.561
Moderate/severe RV dilatation	1.46 (1.02–2.44)	**0** **.** **041**	2.61 (1.25–5.41)	**0** **.** **010**	1.42 (1.01–2.37)	**0** **.** **043**	1.50 (0.85–2.64)	0.159
Moderate/severe RV dysfunction	1.29 (0.96–1.74)	0.096	–	–	1.77 (1.19–2.62)	**0** **.** **005**	1.17 (0.78–1.78)	0.470
TR ERO >60 mm^2^	1.07 (0.78–1.49)	0.640	1.16 (0.81–1.64)	0.416	1.12 (0.65–1.94)	0.666	1.07 (0.68–1.68)	0.784
TCG ≥10 mm	1.44 (1.02–2.06)	**0** **.** **043S**	1.47 (1.13–2.21)	**0** **.** **019**	2.32 (1.53–3.54)	**<0** **.** **001**	2.12 (1.33–3.25)	**0** **.** **001**

ERO, effective regurgitant orifice; LA, left atrial; LVEF, left ventricular ejection fraction; NYHA, New York Heart Association; RA, right atrial, RV, right ventricular; TCG, tricuspid coaptation gap; TR, tricuspid regurgitation.

Bold values refers to *p* ≤ 0.05.

### Prognostic factors in VSTR

For patients with a TCG ≥10 mm, clinical factors associated with all-cause mortality in multivariate analysis were male sex (adjusted HR [95% CI]: 2.45 [1.36–5.17], *p* = 0.004), NYHA class III–IV (adjusted HR [95% CI]: 2.37 [1.15–4.87], *p* = 0.019), and a Charlson comorbidity index >2 (adjusted HR [95% CI]: 1.85 [1.07–3.80], *p* = 0.044), whereas those associated with cardiovascular mortality were male sex (adjusted HR [95% CI]: 2.24 [1.08–4.68], *p* = 0.030), NYHA class III–IV (adjusted HR [95% CI]: 2.25 [1.03–4.88], *p* = 0.042), and ascites (adjusted HR [95% CI]: 2.21 [1.05–4.99], *p* = 0.041) (Table 3).

**Table 3:** Clinical factors associated with all cause and cardiovascular mortality in patients with a tricuspid coaptation gap ≥10 mm.

Variable	All-cause mortality				Cardiovascular mortality			
	Univariable analysis HR (CI[95%])	*p*-value	Multivariable analysis HR (CI[95%])	*p*-value	Univariable analysis HR (CI[95%])	*p*-value	Multivariable analysis HR (CI[95%])	*p*-value
Age >75 years	1.55 (1.12–2.99)	**0** **.** **041**	1.47 (0.81–2.84)	0.202	1.42 (0.69–2.91)	0.341	–	–
Male sex	2.08 (1.10–3.93)	**0** **.** **010**	2.45 (1.36–5.17)	**0** **.** **004**	1.81 (0.90–3.64)	0.095	2.24 (1.08–4.68)	**0.030**
NYHA (3–4 vs. 1–2)	2.64 (1.34–5.23)	**0** **.** **005**	2.37 (1.15–4.87)	**0** **.** **019**	2.54 (1.20–5.36)	**0** **.** **014**	2.25 (1.03–4.88)	**0.042**
Right heart failure	1.45 (0.56–3.76)	0.440	–	–	1.21 (0.46–3.18)	0.706	–	–
Ascites	1.89 (1.17–4.00)	**0** **.** **034**	1.70 (0.75–3.89)	0.204	2.48 (1.14–5.39)	**0** **.** **022**	2.21 (1.05–4.99)	**0.041**
Charlson comorbidity index >2 (without age)	2.01 (1.16–4.04)	**0** **.** **039**	1.85 (1.07–3.80)	**0** **.** **044**	1.51 (0.73–3.14)	0.269	–	–
Atrial fibrillation	1.45 (0.49–4.30)	0.501	–	–	1.18 (0.39–3.59)	0.763	–	–
Diuretics dose ≥125 mg/day	2.25 (1.19–4.25)	**<0** **.** **001**	1.33 (0.62–2.71)	0.479	2.37 (1.17–4.79)	**0** **.** **016**	1.56 (0.71–3.38)	0.263

NYHA, New York Heart Association.

Bold values refers to *p* ≤ 0.05.

For patients with TCG ≥ 10mm, among echocardiographic parameters, only moderate to severe RV dilatation (adjusted HR [95% CI]: 2.61 (1.25–5.41); *p* = 0.010) was associated with all-cause mortality in multivariate analysis. A LVEF between 50% and 60% (adjusted HR [95% CI]: 2.30 [1.09–4.84], *p* = 0.028) and moderate or severe RV dilatation (adjusted HR [95% CI]: 3.61 [1.60–8.13], *p* = 0.002) were independently associated with cardiovascular mortality (Table 4).

**Table 4:** Echocardiographic factors associated with all cause and cardiovascular mortality in patients with a tricuspid coaptation gap ≥10 mm.

Variable	All-cause mortality				Cardiovascular mortality			
	Univariable analysis HR (CI[95%])	*p*-value	Multivariable analysis HR (CI[95%])	*p*-value	Univariable analysis HR (CI[95%])	*p*-value	Multivariable analysis HR (CI[95%])	*p*-value
LVEF 50%–60%	1.73 (0.92–3.27)	0.089	1.71 (0.90–3.26)	0.103	1.92 (0.95–3.68)	0.070	2.30 (1.09–4.84)	**0.028**
Low flow	1.33 (0.70–2.51)	0.387	–	–	1.34 (0.66–2.72)	0.410	–	–
LA area >30 cm^2^	1.39 (0.73–2.63)	0.317	–	–	1.17 (0.58–2.37)	0.664	–	–
RA area >30 cm^2^	1.55 (0.81–2.97)	0.186	–	–	1.70 (0.84–3.47)	0.142	–	–
TR ERO >60 mm^2^	0.62 (0.32–1.19)	0.151	–	–	0.56 (0.28–1.14)	0.110	–	–
Moderate/severe RV dilatation	2.46 (1.19–5.08)	**0** **.** **015**	2.61 (1.25–5.41)	**0.010**	2.88 (1.39–6.27)	**0** **.** **008**	3.61 (1.60–8.13)	**0.002**
Moderate/severe RV dysfunction	1.19 (0.63–2.23)	0.597	–	–	1.41 (0.70–2.83)	0.338	–	–

ERO, effective regurgitant orifice; LA, left atrial; LVEF, left ventricular ejection fraction; RA, right atrial, RV, right ventricular; TR, tricuspid regurgitation.

Bold values refers to *p* ≤ 0.05.

## Discussion

The prevalence of VSTR in patients with significant isolated functional TR was variable, ranging from 17% to 35% of patients, depending on the parameter used to define it. In our population of significant TR, the correlation between EROA and TCG was poor and decreased with increasing TCG. Risk stratification was significantly improved using the TCG but not EROA. Indeed, after adjustment for established prognostic factors, a TCG ≥10 mm was associated with a >2.1-fold increase in the relative risk of cardiovascular death, whereas an EROA ≥60 mm^2^ was not associated with excess mortality. Therefore, a TCG ≥10 mm should be used to define VSTR in isolated functional TR.

The latest European guidelines mentioned that a new grading scheme that includes massive and torrential TR has been proposed by certain authors (10), but to date, there is no official recommendation on the interest of using this classification nor on the definition of “more-than-severe” TR (10). Definitions of massive TR and torrential TR using the EROA and vena contracta have been proposed in the context of transcatheter tricuspid valve repair or replacement (24). Indeed, in the first studies assessing the efficacy of transcatheter tricuspid valve therapies or compared these therapies with medical treatment, TR reduction was only moderate, often leaving patients with significant residual TR (9, 25). In a population of patients with a baseline TR EROA of 0.85 ± 0.22 mm^2^, the mean residual EROA 30 days after treatment was 0.63 ± 0.29 mm^2^ (9). Thus, according to the current classification of the guidelines, these patients would not change in severity grade because they started from a grade of severe TR and still had severe TR after treatment (10, 19–21). However, a significant improvement was observed in terms of stroke volume and quality-of-life scores in these studies (9, 25), highlighting the need to revise TR severity grading. Consequently, it has been proposed to add the massive and torrential grades to the TR classification grading scheme using empirical thresholds for vena contracta and the EROA (11). However, although the current classification is undeniably useful for the assessment of the results of percutaneous treatment, the inclusion of two additional grades has not yet demonstrated its usefulness in the risk stratification of these patients. Indeed, no study has found a difference between massive and torrential forms in terms of outcome prediction (13–15). However, there is now growing evidence (13–15) that adding an additional grade allows risk stratification of all patients with TR (not only those undergoing percutaneous treatment).

Both the EROA and vena contracta present certain limitations. First, interobserver agreement is poor in mitral regurgitation (26, 27) and these parameters are even more difficult to assess in TR because of the particular and variable geometry of this valve and the fact that it is often difficult to be perpendicular to the jet to accurately measure vena contracta (16). It should be noted that although a number of reports have validated thresholds of EROA >40 mm^2^ (28) and EROA is a reliable parameter to identify severe vs. non-severe TR with numerous robust data reported (8, 17, 19, 20, 29). Nevertheless, it may not be well suited for the most severe cases of TR. Indeed, the use of EROA assessed by the PISA method relies on geometric hypotheses, which are true for primary mitral regurgitation but not necessarily for TR, in particular, very severe functional secondary TR. Indeed, most PISA assumptions are often not respected in secondary very severe TR, as there is temporal and respiratory variability in TR, the flow convergence zone is rarely hemispheric, and the surface is not flat, as tethering of the leaflets is often present. Therefore, the PISA method faces several limitations in VSTR and could significantly underestimate the severity of TR by up to 30% (30, 31). With increasing coaptation defect, the geometry of the orifice becomes less circular and therefore the PISA becomes less reliable. Accordingly, in our population, the relationship between the TCG and EROA was poor (*R*^2 ^=^ ^0.21), especially for patients with a large TCG. Indeed, among patients with a TCG ≥10 mm, those with the lowest EROA (<40 mm^2^) had the largest TCG (mean 17.9 mm). Conversely, TCG measurements are relatively easy to perform when TCGs are present. We found a better reproducibility for the TCG measurement than for the EROA probably because the TCG requires only one measurement whereas the calculation of the EROA by the PISA technique requires several parameters (PISA radius, *V*max, VTI), which increases the risk of error and is more technically challenging to obtain. Thus, to summarize, the shape of the regurgitant orifice as well as the frequent not flat surface due to the tethering of the leaflets and the fact that the PISA method is less reliable due to greater variability are our main hypotheses to explain the superiority of TCG over PISA EROA in very severe TRs. Rapid developments in three-dimensional transthoracic echocardiography will soon allow routine measurement of anatomical EROA, which will certainly be more accurate because it will assess the true coaptation defect in all three dimensions but will require clinical validation. Pending the widespread adoption of these new techniques, the two-dimensional measurement in end-systole in the view where the TCG is the largest is simple and present good interobserver reproducibility (*r* = 0.93, intraclass correlation coefficient = 0.96 [0.86–0.99]) (14). We found, in our study population of significant TR, that the two-dimensional measurement of TCG was better than the EROA for patient risk stratification, which was markedly improved for a TCG ≥10 mm but not an EROA ≥60 mm^2^. Therefore, a TCG ≥10 mm should be used to define VSTR.

### Limitations

Our study had several limitations. First, it was retrospective and suffered from the inherent limitations of this type of analysis. Echocardiograms were not analysed in a central corelab but at each institution separately. The assessment of RV dimensions is challenging due to its complex geometry and the lack of specific right-sided anatomic landmarks to be used as reference points. Unfortunately, we did not dispose of right ventricular surface or 3-dimensional volume data which is a limitation and, in our study, RV dilatation was defined by a diameter >42 mm. The definition of RV dysfunction was based on TAPSE and *S*′ measurements, despite the limitations of these parameters in the presence of severe TR. Nevertheless, although normal RV systolic function cannot be determined with certainty when the values are normal, the presence of a dysfunction is clear when these parameters are altered. While the extent of TCG is a reflect of the anatomic ERO, TCG might also reflect RV enlargement and/or dysfunction and tricuspid annular dilatation especially when RV dilatation contributes significantly to the severity of TR. However, in isolated functional (or atrial) TR, RV dilatation is not the cause of the disease but rather a late consequence of the regurgitation (32, 33). Actually, TCG in our study remained independently associated with all-cause and cardiovascular mortality after adjustment for RV dilatation and dysfunction and the relationship between TCG and basal RV diameter and between TCG and tricuspid annular diameter were weak (*R*^2 ^=^ ^0.17 and 0.19 respectively). We did not include the laminar character of the TR and the inversion of the hepatic vein flow in the definition of VSTR, contrary to our previous study (14) because we had some missing data for these parameters and we wanted to propose a simple definition. In patients with these parameters available (82% for the laminar character of the flow and 77% for the hepatic vein flow), they were always present in case of TCG ≥ 10 mm (Table 1). We believe that these are both important criteria that should be investigated systematically for the quantification of TR. Unfortunately, data on aliasing velocity (*V*_a_) were not collected in our database and we cannot multiply the calculated flow rate by *V*_max_/(*V*_max_ − *V*_a_) to yield the correct regurgitant flow rate (34). However, to avoid this underestimation, the ratio of the aliasing velocity to the tricuspid regurgitant jet velocity in our study was maintained, as recommended, <10% whenever feasible (92% of patients) (20). The TCG presence indicates a significant TR and cannot be used for the quantification of mild to moderate forms. Measurement of the TCG may be challenging after percutaneous tricuspid valve edge-to-edge repair, but this is also true of the PISA and vena contracta methods, which have never been validated in these patients. Finally, our results are only valid for patients with isolated functional TR and may not be applicable to other TR etiologies. Indeed, we chose to study isolated TR to have a homogeneous group and evaluate the true impact of the severity of TR on the prognosis, without interference from other valvular heart diseases, pulmonary hypertension, or left ventricular dysfunction, which could have affected the outcome and biased the results.

## Conclusions

The correlation between the TCG and the EROA is weak in significant TR and decreases with increasing defect size due to limitations inherent to the PISA technique. A TCG ≥10 mm is associated with a significant increase in all-cause and cardiovascular mortality and should be used to define VSTR in isolated significant functional TR.

## Data Availability

The raw data supporting the conclusions of this article will be made available by the authors, without undue reservation.
