# Supplementary material for: Comparison of effective regurgitant orifice area by the PISA method and tricuspid coaptation gap measurement to identify very severe tricuspid regurgitation and stratify mortality risk
Source: Front Cardiovasc Med. 2023 Apr 27;10:1090572. doi: 10.3389/fcvm.2023.1090572 (PMC10172668; doi:10.3389/fcvm.2023.1090572)

<u>Supplemental Figure 1:</u> Receiver operating characteristic curve (ROC) curve analysis of the best parameters for predicting very severe TR

AUC: area under the curve; EROA: effective regurgitant orifice area; TCG: tricuspid coaptation gap; TR: tricuspid regurgitation.

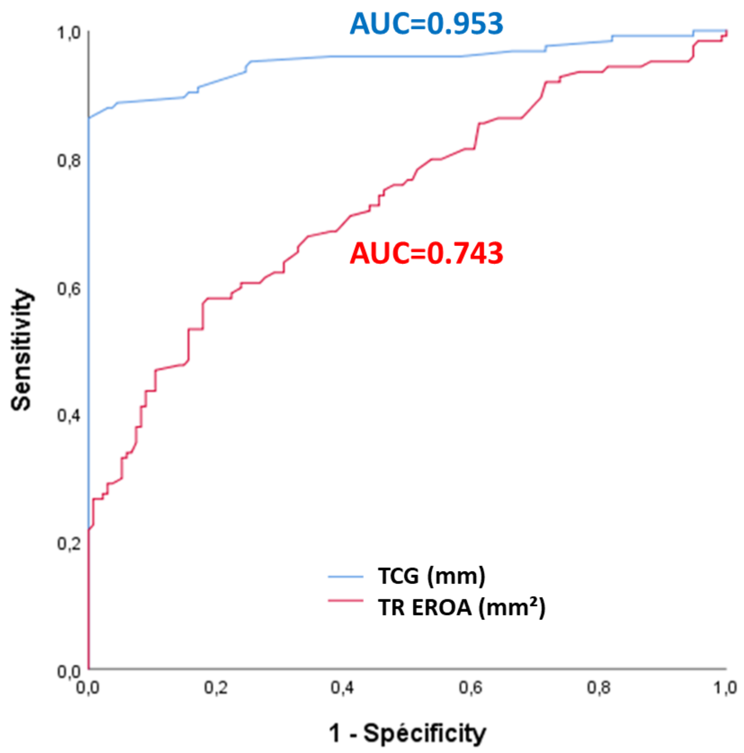

Supplement: Supplementary file 1 [file Datasheet1.pdf]
